# Hemagglutinin E190D substitution in clade 2.3.4.4b A(H5N1) influenza virus reduces receptor binding and viral fitness

**DOI:** 10.1038/s44298-026-00197-2

**Published:** 2026-05-15

**Authors:** Xiangjie Sun, Claudia Lisboa, Paul J. Carney, Jessie C. Chang, Brandon L. Bradley-Ferrell, Xiao-yu Zheng, Jessica A. Belser, Nicole Brock, Troy J. Kieran, Hui Zeng, Joanna A. Pulit-Penaloza, Rebecca J. Kondor, James Stevens, Taronna R. Maines

**Affiliations:** Centers for Disease Control and Prevention, Atlanta, GA USA

**Keywords:** Genetics, Microbiology

## Abstract

The detection of a clade 2.3.4.4b influenza A(H5N1) virus bearing an HA-E190D substitution in an infected human raises concern about mammalian adaptation. We evaluated the impact of the naturally occurring HA-E190D substitution in the A/British Columbia/PHL-2032/2024 (BC/24) virus on receptor binding specificity and viral fitness in vitro and in vivo. Recombinant BC/24 HA-E190D protein retained α2,3-linked sialic acid binding specificity but with reduced binding affinity. In cell culture, BC/24 viruses dominated by HA-190D were frequently outcompeted by the minor HA-190E variant in the inoculum, whereas BC/24 HA-190E viruses maintained dominance in the presence of residual HA-190D in the inoculum. In ferrets, BC/24 HA-190D viruses were similarly outcompeted by HA-190E; in one out of six ferrets where HA-190D dominance persisted, the virus was attenuated and failed to spread systemically. In contrast, BC/24 HA-190E viruses maintained dominance and disseminated systemically. These results indicate that the BC/24 variant bearing HA-E190D substitution did not acquire human-like receptor binding specificity and was less fit in mammalian hosts, making it unlikely to enhance public health risk without additional compensatory mutations.

## Introduction

Since 2020, highly pathogenic avian influenza (HPAI) A(H5N1) clade 2.3.4.4b viruses have spread rapidly worldwide with concurrent spillovers in over 40 mammalian species^1^. This clade of A(H5) virus was first detected in the United States in late 2021, and has since caused thousands of outbreaks in poultry and dairy cattle, along with 72 confirmed human A(H5) cases in the United States, including two fatal cases, as of November 2025^2–4^. The expanded host range and ongoing evolution of clade 2.3.4.4b A(H5N1) viruses highlight the urgent need for coordinated global surveillance and close monitoring of viral evolution dynamics, particularly the emergence of variants (referring to viruses that differ genetically or antigenically from reference strains due to acquired amino acid changes during circulation) with enhanced potential for mammalian adaptation and transmission.

A growing number of amino acid substitutions that facilitate the adaptation and airborne transmission of avian-origin influenza A viruses (IAVs) in mammalian hosts have been identified^5^. Among these, key substitutions in the hemagglutinin (HA) receptor binding site that shift receptor binding specificity from α2,3-linked sialic acids (avian-like receptors) to α2,6-linked sialic acids (human-like receptors), as well as mutations in the polymerase complex—such as E627K in the polymerase basic protein 2 (PB2), which facilitates virus replication in mammalian hosts—have been shown to play critical roles in the emergence of previous pandemic influenza viruses^6^. It has been postulated that other avian-origin IAV subtypes may follow similar evolutionary pathways to acquire sufficient replication capacity and airborne transmissibility in mammalian hosts, potentially leading to a pandemic. Consequently, there has been a strong emphasis on molecular surveillance of IAVs to detect strains or variants harboring mutations associated with mammalian adaptation^7^.

In November 2024, an A(H5N1) clade 2.3.4.4b virus (A/British Columbia/PHL-2032/2024, genotype D1.1) was isolated from the tracheal aspirate collected from an individual with severe disease. Sequencing confirmed variants carrying E190D (mature H3 numbering used throughout, 186 in mature A(H5) numbering, 28% frequency) and Q226H (222 in mature A(H5) numbering, 35% frequency) substitutions in HA, as well as an E627K substitution in PB2 (52% frequency)^8^. In addition, a human A(H5N1) isolate from a fatal case in Louisiana (A/Louisiana/1/2024, genotype D1.1) was found to contain the HA-E190D variant at a frequency of 8% in a combined nasopharyngeal and oropharyngeal sample^9^. The D1.1 genotype is different from the B3.13 genotype, causing widespread outbreaks in dairy cattle in the United States. The emergence of the HA-E190D variant in the A(H5) subtype of IAV has raised concerns because prior studies have shown that the HA-E190D substitution, along with the Q225D mutation, enables a shift from avian- to human-like receptor binding specificity in A(H1N1) viruses and was a key prerequisite for the emergence of the 1918 pandemic influenza virus^10,11^. Although the HA-E190D substitution in a clade 1A(H5N1) virus did not switch receptor binding specificity^12^, whether such a mutation confers enhanced potential for mammalian adaptation in clade 2.3.4.4b A(H5N1) viruses remains uncertain.

In this study, we focused on assessing the impact of the HA-E190D mutation in a clade 2.3.4.4b A(H5N1) human isolate on viral receptor binding specificity, replication, and fitness both in vitro and in vivo. The findings from this study hold significant implications for pandemic preparedness and informing surveillance strategies.

## Results

### Rare occurrence of HA-190D in A(H5) subtype IAVs

The detection of the HA-190D variant in the BC/24 virus, isolated from a severe human case, prompted us to investigate the prevalence of this substitution among the A(H5) subtype of IAVs. A(H5) HA sequence was retrieved from the GISAID database^13^ and analyzed. HA sequence alignment revealed that HA-190E is highly conserved across all examined HA sequences (44,245 of 44,505 HA sequences, 99.4%) (Supplementary Table 1) regardless of host origins, and propagation history (either from eggs or cells). Additional amino acid substitutions at this position include valine (V; 119 sequences), glycine (G; 22 sequences), lysine (K; 5 sequences), aspartic acid (D; 3 sequences), and single occurrences of histidine (H), isoleucine (I), asparagine (N), proline (P), glutamine (Q), serine (S), and threonine (T). The three additional A(H5) HA sequences with HA-190D were from A/mallard/Bavaria/41/2006 (EPI_ISL_68243; Eurasian lineage, non A/Goose/Guangdong clade)^14^, A/Egypt/N00002/2011 (EPI_ISL_120310; clade 1)^15^, A/Vietnam/CL01/2004 (EPI_ILS_9825; clade 2.2.1.2)^16^. The rarity of the HA-190D among A(H5) sequences in original specimens from both avian and mammalian hosts suggests that this residue likely confers a limited fitness advantage under natural selective pressures.

### BC/24 HA-190D variant retained avian-like receptor binding specificity but exhibited reduced binding affinity and no change in the pH of fusion activation

**Fig. 1:**
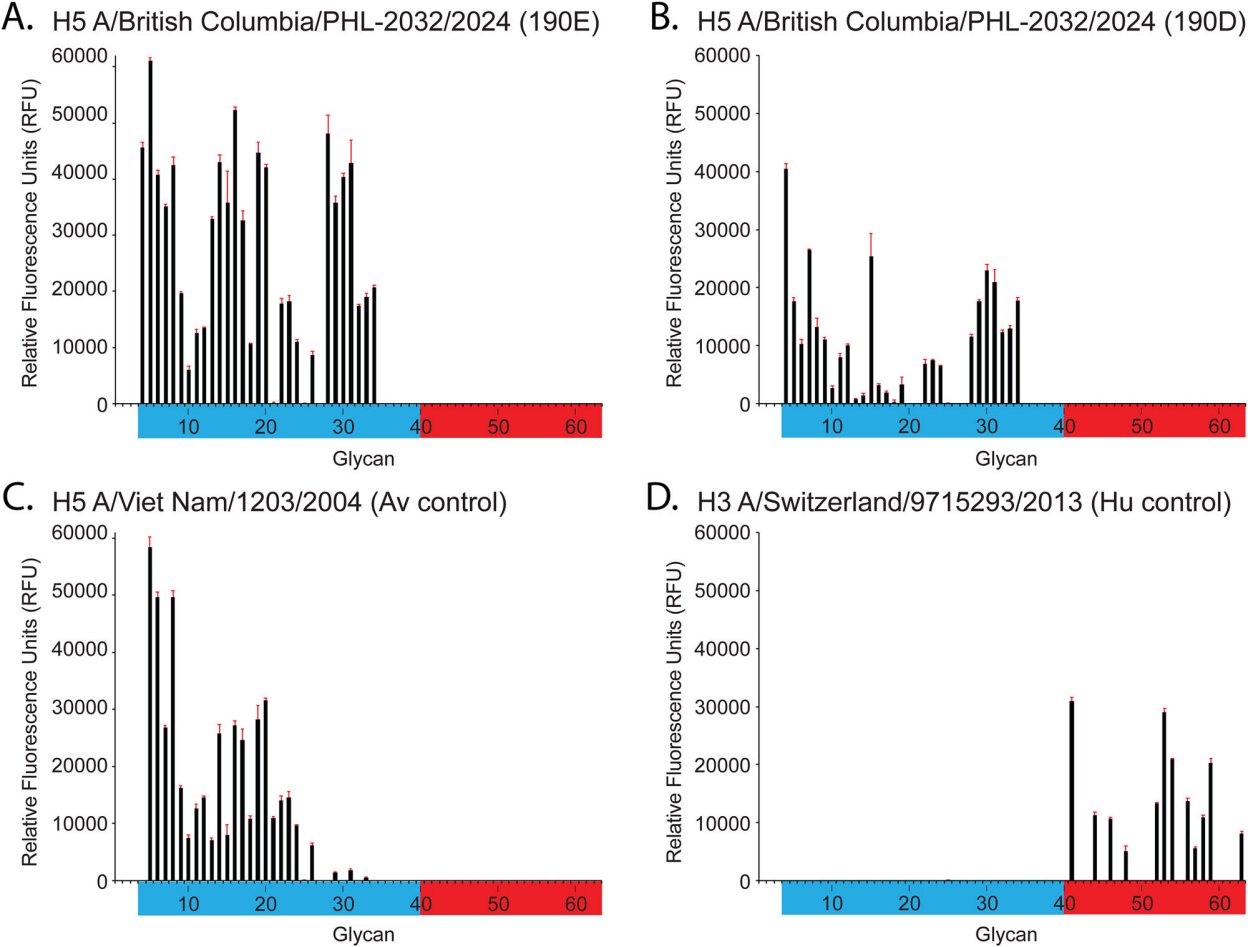
Glycan microarray analyses. Hemagglutinin proteins derived from A/BC/24 HA-190E (**A**), A/BC/24 HA-190D (**B**), A/Viet Nam/1203/2005 A(H5N1) (control for avian-like receptor binding; Av control) (**C**), and A/Switzerland/97/15293/2013 A(H3N2) (control for human-like receptor binding; Hu control) (**D**) were processed for glycan microarray analyses. Glycans marked in blue (#1–40) and red (#41–63) in the X-axis represent α2,3-linked sialic acids (avian-like) and α2,6-linked sialic acids (human-like), respectively. Error bars are standard deviations from six independent replicates on the array. The structures of each of the numbered glycans are listed in Supplementary Table 1.

Because the HA-E190D substitution has previously been shown to switch receptor binding specificity from α2,3-linked (avian-like) to α2,6-linked (human-like) sialic acids in A(H1N1) viruses^11,17^, we first evaluated whether this substitution alters receptor binding specificity or affinity in the clade 2.3.4.4b A(H5N1) virus. Recombinant HA proteins derived from the BC/24 virus bearing either residue 190E or 190D were examined using a glycan binding assay, along with the control HA proteins from A/Viet Nam/1203/2004 (Clade 1, A(H5N1); control for avian-like receptor binding) and A/Switzerland/97/15293/2013 (A(H3N2); control for human-like receptor binding). As shown in Fig. 1, the BC/24 HA-190E protein bound exclusively to α2,3-linked sialic acids (Fig. 1A, #1–40). The BC/24 HA-190D protein did not exhibit any binding to the human receptor analogs present on the array (Fig. 1B, #41–63). Indeed, the recombinant BC/24 HA-E190D protein showed a reduced affinity to α2-3 sialosides. Linear glycans (Fig. 1B, #13–20) were markedly reduced in their binding, while sulfated (Fig. 1B, #4–8) and fucosylated glycans (Fig. 1B, #28–34) still maintained significant binding. These results are consistent with previous studies that showed the HA-E190D substitution on the A/Viet Nam/1203/2004 framework was insufficient to confer a switch from avian-like to human-like receptor binding^12^.

Substitutions in the HA receptor binding site can potentially impact the pH thresholds for HA activation, an important biological property that has been implicated in viral pathogenicity and airborne transmissibility in mammalian hosts^18^. However, both BC/24 HA-190E and HA-190D viruses maintained a fusion activation pH of 5.7 as evaluated by a syncytium formation assay (Supplementary Fig. 1), which is in the range of values reported for other A(H5) viruses from this clade^19,20^, indicating that the BC/24 HA-190D variant has not yet acquired the essential characteristics for mammalian adaptation in the HA.

### BC/24 HA-190D and HA-190E viruses exhibited differing fitness in vitro

**Fig. 2:**
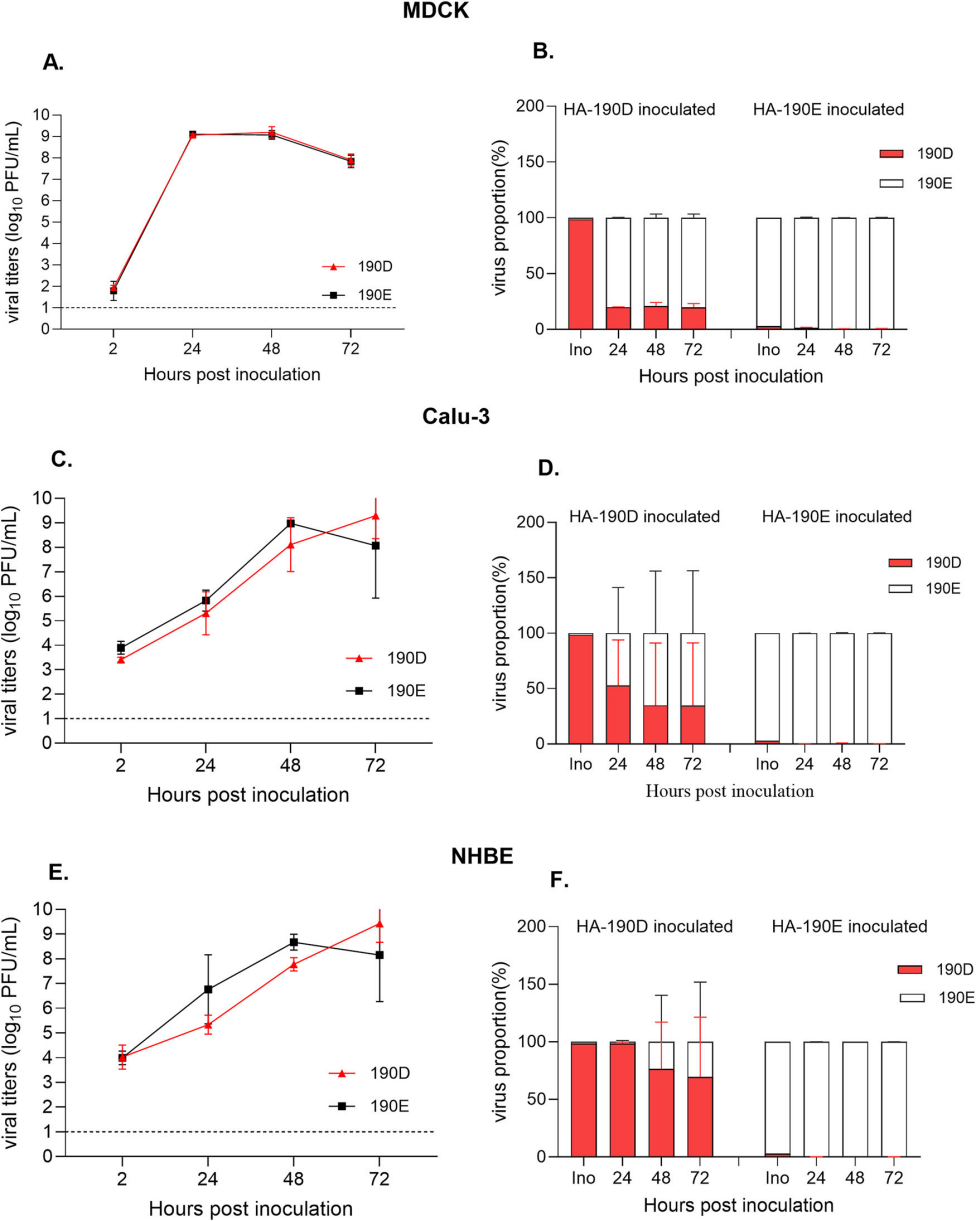
Replication kinetics and frequencies of BC/24 HA-190D and HA-190E variants in MDCK, Calu-3, and NHBE cells. MDCK cells were cultured in 6-well plates, and Calu-3 and NHBE cells were cultured in 12-well plates with Transwell inserts under air-liquid interface conditions, subsequently cells were inoculated with either BC/24 HA-190D (98.6% frequency) or HA-190E (97.1% frequency) virus at an MOI of 0.01. Cell culture supernatants were collected at 2, 24, 48, and 72 h p.i. and viral titers were determined by standard plaque assay in MDCK cells. Mean viral titers with standard deviations from three biological replicates are shown with a solid connecting line for each virus across 72 h p.i. (**A**, **C**, **E**). Limits of detection are indicated with dotted horizontal lines. Differences in viral titers between BC/24 HA-190D and HA-190E virus at 2, 24, 48, and 72 h p.i. in each cell type were analyzed for statistical significance using two-way ANOVA followed by Sidak’s multiple comparisons test in GraphPad Prism 10.5.0, and no statistically significant differences were observed between the two viruses at any time point. Additionally, the mean frequencies of the BC/24 HA-190D and190E variants in supernatants collected from the inoculated cells along with the inoculum (Ino) were quantified by next-generation sequencing (NGS) and are presented along with SD in bar graphs (**B**, **D**, **F**). Variant calling was performed in Geneious Prime (Version 2020.2.4) using a 1% frequency threshold and a minimum sequencing-depth cutoff of 30×.

As the HA-190 position is in a key region modulating receptor binding specificity and has the potential to influence viral replication in host cells, we first compared viral replication kinetics of BC/24 viruses predominantly containing either the HA-190D (98.6% frequency) or HA-190E (97.1% frequency) variant in three different cell types. These include primary normal human bronchial epithelial (NHBE) cells and an immortalized human airway epithelial cell line (Calu-3), which emulate the physiological sites for viral replication in the human upper and lower respiratory tract, respectively, and MDCK cells, which are commonly used for IAV stock propagation. Our results showed that both viruses exhibited efficient replicative capacity and reached peak titers of approximately 10^9^ PFU/mL or higher for all cell types (Fig. 2A, C, E). Notably, viral replication in MDCK cells was particularly robust and consistent between the two viruses, with titers exceeding 10^9^ PFU/mL as early as 24 h post inoculation (p.i.) (Fig. 2A). In contrast, in both Calu-3 and NHBE cells, BC/24 HA-190E and BC/24 HA-190D viruses did not reach peak titers until 48–72 h p.i. (Fig. 2C, E). Although both viruses reached comparable peak titers, the BC/24 HA-190D virus exhibited slightly lower early replication than BC/24 HA-190E at 24–48 h p.i. in NHBE and Calu-3 cells; however, this difference did not reach statistical significance (Fig. 2C, E).

Next, we employed next-generation sequencing (NGS) to determine whether each viral species maintained its predominance during replication in vitro. We observed that in all three cell types inoculated with the BC/24 HA-190D virus, containing a minor HA-190E variant at ~1.4% frequency, the predominance of the HA-190D virus decreased relative to the inoculum by 72 h p.i. Specifically, in NHBE cells, the HA-190D virus initially remained highly dominant (>98%), comparable to the original stock virus at 24 h p.i., but gradually declined to ~70% by 72 h p.i. (Fig. 2F). In Calu-3 cells, the frequency of the HA-190D variant declined to ~53% at 24 h p.i. and further decreased to ~35% by 48 and 72 h p.i. (Fig. 2D). In MDCK cells, the frequency of the HA-190D variant remained consistently low, at ~20%, throughout the 72 h replication period (Fig. 2B). In all three cell types, the reduced frequency of the HA-190D in the cell culture supernatants was compensated by the increased presence of the HA-190E, and no other variants at this position were detected.

In contrast, in all three cell types inoculated with the BC/24 HA-190E virus, containing residual HA-190D variant (2.9% frequency), the HA-190E virus exhibited a marked growth advantage, maintaining nearly 100% frequency throughout the 72 h replication period; the minor HA-190D variant present in the inoculum was almost completely diminished (Fig. 2B, D, F). Taken together, these findings indicate that the BC/24 HA-190D variant has a growth disadvantage in cell culture, with a tendency to be outcompeted by HA-190E during multiple rounds of replication. Nevertheless, the BC/24 HA-190D virus appears capable of maintaining partial predominance in certain cell types, such as NHBE. In contrast, the BC/24 HA-190E virus exhibited substantial fitness in all tested cell types.

### BC/24 HA-190E virus exhibited efficient replication and is capable of systemic spread in ferrets

We next evaluated the replication and in vivo fitness of the BC/24 HA-190E virus in the ferret model, which is widely regarded as the most physiologically relevant animal model for assessing IAV pathogenicity and transmission^21^. Ferrets that were intranasally inoculated with 100 PFU of the BC/24 HA-190E stock virus exhibited clinical signs including lethargy, nasal discharge, and fever. The inoculated ferrets experienced an average body weight loss of 6% by day 3 p.i. and a mean maximum temperature increase of 1.5 °C above baseline. Ferrets were humanely euthanized on day 3 or day 4 p.i. (representative timepoints of peak viral replication in ferrets) to assess viral variant dynamics following multicycle replication.

**Fig. 3:**
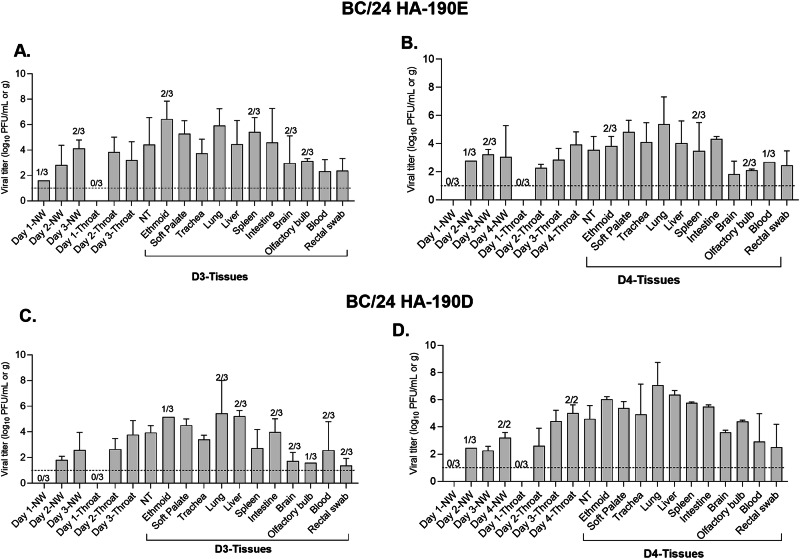
Replication and systemic spread of the BC/24 HA-190E and HA-190D virus in ferrets. Six naïve ferrets were intranasally inoculated with 100 PFU of the BC/24 HA-190E (**A**, **B**) or HA-190D (**C**, **D**) virus. Three ferrets were humanely euthanized for necropsy on days 3 and 4 p.i. for each virus group, except that there were only two ferrets in the BC/24 HA-190D inoculated group on day 4, as one ferret succumbed to infection prior to the scheduled necropsy. Infectious viral titers in collected nasal washes (NW), throat and rectal swabs, blood, and tissues were determined by standard plaque assay. The limit of detection (10 PFU/mL or gram (g)) is indicated by a dashed horizontal line. Bar graphs represent viral titer means, excluding samples below the detection limit, expressed as log10 PFU per mL (for fluids) or g (for tissues) with standard deviations. When infectious viruses were not detected in all three animals in that group, the number of ferrets with detectable virus is indicated above the corresponding bars. Differences in viral titers in each specimen type between ferret groups inoculated with BC/24 HA-190D or HA-190E virus on days 3 and 4 were analyzed for statistical significance using two-way ANOVA followed by Sidak’s multiple comparisons test in GraphPad Prism 10.5.0.0, including only datasets in which the virus was detected in all 3 inoculated animals per group. Day 4 data shown in Fig. 3B, D were excluded from analysis because only two ferrets remained in the BC/24 HA-190D inoculated group at that time point. No statistically significant differences were observed between the two groups across any specimen type (*p* > 0.05).

Infectious virus was detected in all six ferrets inoculated with the BC/24 HA-190E virus as early as day 2 p.i. in throat swabs, with viral titers reaching ~10^3^ PFU/mL by day 3 p.i. (Fig. 3A, B). Viral titers in nasal washes were comparable to those observed in the throat swabs, with mean viral titers of 10^2.8^ and 10^3.7^ PFU/mL on days 2 and 3, respectively, among ferrets with detectable infectious viruses. Additionally, despite the low inoculum dose (100 PFU), the BC/24 HA-190E virus exhibited efficient dissemination, reaching both respiratory and extrapulmonary tissues by days 3 or 4 p.i. Viral titers in respiratory tissues exceeded 10^3.5^ PFU/g, and similar titers were observed in the liver and intestines. The BC/24 HA-190E virus was also detected at levels of ~10^2.0^ PFU/g or mL or higher in most blood, brain, and olfactory bulb tissue specimens, demonstrating systemic viral spread (Fig. 3A, B). For all nasal wash, throat swab, and tissue samples that were successfully sequenced, only the HA-190E genotype was detected, with no emergence of the HA-190D variant (at the 1% threshold) at the HA 190 position, or other major nonsynonymous variants (≥15% frequency) across the viral genome, indicating a robust fitness advantage of the BC/24 HA-190E virus during replication in ferrets.

### BC/24 HA-190D virus was frequently outcompeted by HA-190E in ferrets

**Fig. 4:**
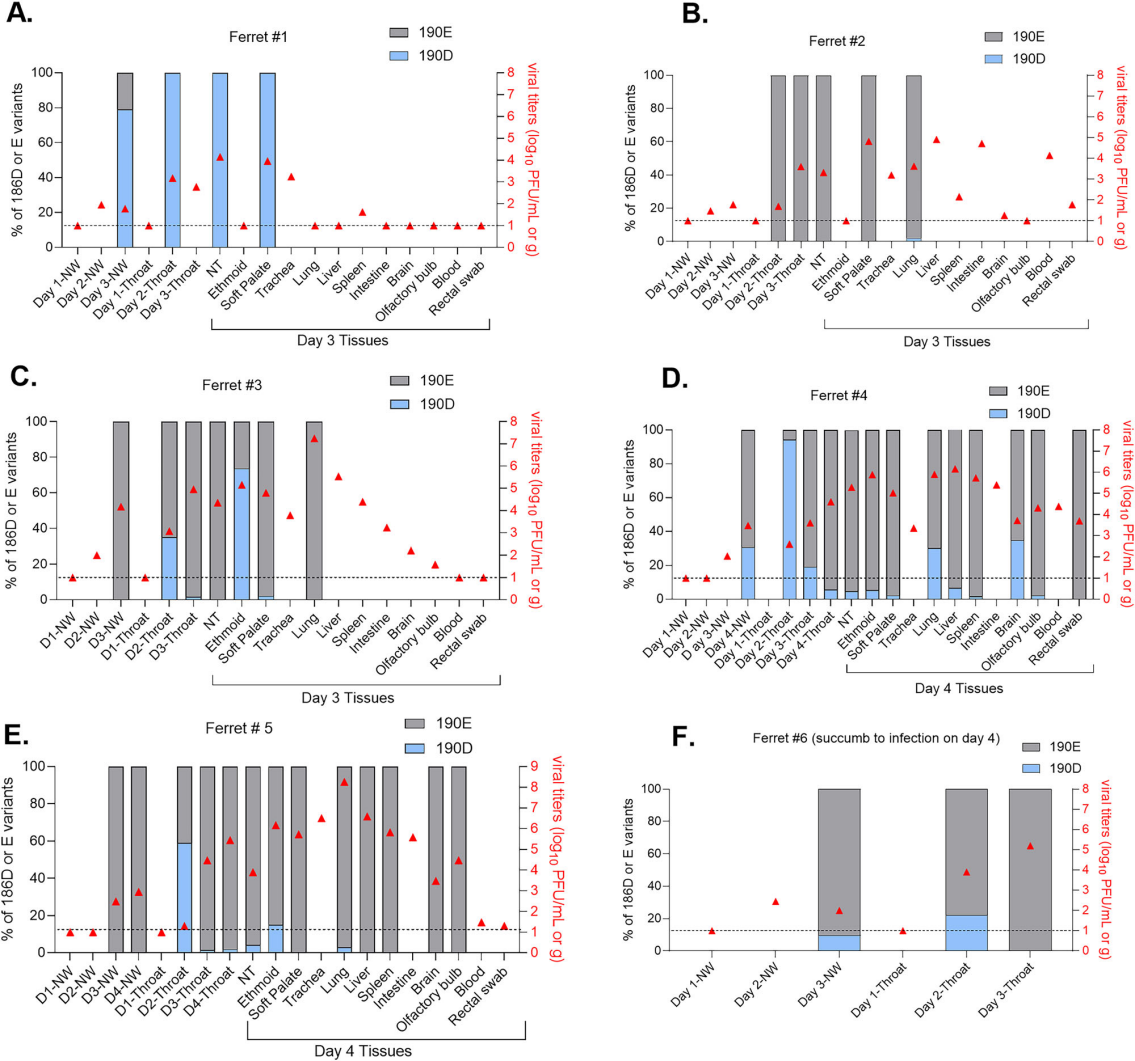
Replication, systemic spread, and variant population dynamics of the BC/24 HA-190D virus in ferrets. Six naïve ferrets (showing individually in **A**, **B**, **C**, **D**, **E**, **F**) were intranasally inoculated with 100 PFU of the BC/24 HA-190D virus. Three ferrets each were humanely euthanized for necropsy on days 3 and 4 p.i. Infectious viral titers in the nasal washes (NW), throat and rectal swabs, blood, and tissues collected from individual ferrets were determined by standard plaque assay and are indicated by the red triangles, with the right Y-axis showing viral titers expressed as log10 PFU per mL (for fluids) or g (for tissues). The limit of detection (10 PFU/mL or g) is indicated by a dashed horizontal line. The frequencies of HA 190D and 190E variants in select specimens, which were sequenced and achieved a minimum sequencing depth of >30× by the Illumina NGS platform, are displayed as a bar graph, with the left Y-axis scaled to 100%.

We next applied the same experimental approach to assess the pathogenicity, replication, and in vivo fitness of the BC/24 HA-190D virus in the ferret model. Ferrets inoculated with the BC/24 HA-190D virus exhibited clinical signs similar to those inoculated with the BC/24 HA-190E virus, including comparable mean weight loss (5.8%) and peak temperature rise (1.9% above baseline). Analysis of viral dissemination in nasal and throat specimens, as well as in tissues, showed that mean viral titers among animals with detectable viruses on days 3 and 4 p.i. were not significantly different from those observed in ferrets inoculated with the BC/24 HA-190E virus (Fig. 3A versus C, B versus D). However, Ferret #1, euthanized on day 3 p.i., displayed limited viral spread, with infectious virus detected almost exclusively in the upper respiratory tract (Fig. 4A). This animal also experienced slightly less body weight loss (3.5% by days 3 p.i.) compared with the group mean (5.8%).

NGS analysis of genetic identity revealed substantial variability in HA-190D predominance among the inoculated animals. Ferret #1 retained an almost exclusively HA-190D population in upper respiratory tract specimens (nasal wash, throat, nasal turbinate, and ethmoid turbinate), except for the day 3 p.i. nasal wash, where the HA-190D frequency decreased to ~80% (Fig. 4A) with the remaining viral population being HA-190E virus. In contrast, Ferret #2 harbored an almost exclusively HA-190E population in both throat swabs and tissues (Fig. 4B). This ferret experienced a maximum weight loss of ~7% and a temperature rise of ~2.3 °C above baseline by day 3 p.i., and virus spread systemically in this ferret, with titers exceeding 10^3^ PFU/g or mL in the nasal turbinate, trachea, lung, liver, intestine, and blood.

The remaining four inoculated ferrets displayed mixed populations containing both HA-190D and HA-190E variants, with HA-190E predominating in nearly all nasal washes and tissues except for a single ethmoid turbinate sample from Ferret #3 (Fig. 4C–F). In throat specimens that were collected on multiple days, the frequency of HA-190D progressively declined over time, demonstrating a selective disadvantage relative to HA-190E. Ferret #6 possessed ~88% HA-190E in the day 2 throat sample, which increased to 100% by day 3 p.i.; this ferret succumbed to infection on day 3 p.i. prior to scheduled necropsy (Fig. 4F).

In conclusion, our results demonstrate that the BC/24 HA-190D virus was less fit in inoculated ferrets. The minor HA-190E variant present in the inoculum rapidly outcompeted the HA-190D population in most animals. In the rare instances where HA-190D predominance was maintained (Ferret #1), the virus displayed an attenuated phenotype, characterized by restricted replication limited to the upper respiratory tract and inability to disseminate to the lower respiratory tract or extrapulmonary tissues.

## Discussion

Early detection and rapid assessment of zoonotic influenza variants with potential for enhanced mammalian adaptation are critical components of pandemic preparedness. In response to the recent detection of a clade 2.3.4.4b A(H5N1) variant bearing an HA-E190D substitution from two human A(H5) cases, a residue previously implicated as a key determinant of receptor binding specificity and pandemic potential in the A(H1N1) 1918 virus, we conducted a comprehensive investigation to assess the impact of this substitution on receptor binding properties and viral fitness in both in vitro and in vivo systems.

A switch from avian- to human-like receptor binding is one of the key prerequisites for avian-origin IAVs to replicate efficiently and transmit in humans^22^. Although clade 2.3.4.4b A(H5N1) viruses have shown expanded mammalian tropism and possible mammal-to-mammal transmission^23,24^, viruses isolated from mammals generally retain a strong avian-like receptor binding preference with minimal α2,6-linked sialic acid binding^20,25–27^. Multiple substitutions in A(H5) HA receptor binding sites, including D187G, E190G/Q/A, K193R, Q196R, N224K/Y, Q226L, S227N, and G228S, can modulate receptor binding specificity in a strain- or clade-dependent manner^28–32^. Notably, in a recent study with clade 2.3.4.4b A/Texas/37/2024 (TX/24) virus, the first human A(H5) case associated with exposure to infected cattle, HA-Q226L alone shifted specificity to human-like receptors, with HA-N224K further enhancing such binding capabilities^33^; however, the impact of such substitutions on viral pathogenicity and transmissibility of clade 2.3.4.4b A(H5N1) virus remains unknown. In this study, we investigated a naturally occurring HA variant in the BC/24 virus from a severe human case of A(H5N1) virus infection. Consistent with observations with TX/24 and other A(H5N1) viruses in clade 2.3.4.4b^20,25^, BC/24 HA with the consensus 190E residue bound exclusively to α2,3-linked sialic acids. The BC/24 HA with 190D reduced α2,3-linked sialic acid binding without acquiring α2,6-linked sialic acid binding capacity, consistent with observations in other H5 HAs^12,29^. Collectively, these findings suggest that HA-E190D alone does not confer human-like receptor binding, although the reduction in α2,3-sialic acid binding may represent a potential intermediate state that could potentiate a shift toward human-like receptor binding when combined with additional mutations. Continuous monitoring of mutations in the HA, including other variants, such as Q227H, present at 35% frequency in the BC/24 clinical isolate, and timely evaluation of the impact of those mutations on influenza A(H5) viral receptor binding is needed.

To further assess fitness consequences of the HA-E190D substitution, we utilized two BC/24 viral stock propagations, each naturally dominated by either the HA-190D or HA-190E variant, to evaluate viral replication and population dynamics in vitro and in vivo. The BC/24 HA-190E virus exhibited a robust fitness advantage in all systems assessed, including efficient replication in multiple cell lines, high-titer replication in the upper respiratory tract of ferrets, and systemic spread to extrapulmonary tissues and blood in inoculated ferrets, a feature typically associated with severe disease. The residual HA-190D variant in the inoculum rapidly became undetectable in both systems, likely reflecting the reduced receptor binding affinity. The observed fitness advantage associated with HA-190E is consistent with a recent study, in which ferrets were inoculated with a high dose (10^6.0^ PFU) of the BC/24 virus containing with 100% HA-190E in the inoculum, which exhibited efficient viral replication and systemic dissemination, and no emergence of HA-190D variant was detected^34^. It is worth noting that the HA-190D variants detected in two human cases infected with clade 2.3.4.4b A(H5N1) viruses from D1.1 genotype were identified in specimens collected during the course of clinical symptom development^8,9^. The same variant was not detected in closely related avian viruses, indicating that the emergence of HA-190D likely occurred during viral replication in humans rather than being directly acquired from avian species.

In contrast, the BC/24 HA-190D virus exhibited reduced fitness in both cell culture and ferret infection models. When evaluated in three different cell types, with NHBE and Calu-3 cells representing the upper and lower respiratory tract, respectively, the residual BC/24 HA-190E variant present in the HA-190D stock tended to outcompete the BC/24 HA-190D virus, with the degree of displacement varying by cell type. These cell-type-dependent differences suggest that specific anatomic niches within mammalian hosts may facilitate the persistence of this variant, allowing it to be maintained despite its overall fitness disadvantage. A similar trend of reduced fitness associated with the HA-190D variant was also observed in vivo. In ferrets inoculated with the BC/24 HA-190D dominant stock, which contained only ~1.4% HA-190E variant, the HA-190D virus typically lost dominance as early as days 2 p.i. and was often completely replaced by the HA-190E variant as infection progressed. In 1 out of 6 inoculated ferrets, HA-190D dominance was retained, likely reflecting stochastic effects following low MOI inoculation; viral replication was, for the most part, restricted to the upper respiratory tissues and soft palate, indicating an attenuated pathogenicity. The distinct phenotypes exhibited by HA-190E and HA-190D viruses highlighted the functional importance of residue 190 in modulating pathogenicity.

Taken together, these results demonstrate that the HA-E190D variant on an A(H5N1) virus backbone is less fit than its HA-190E counterpart and is associated with limited viral spread and reduced pathogenicity in ferrets. Evaluation of the receptor binding and pathogenicity of emerging variants, especially those carrying substitutions within the receptor binding site or key positions in the polymerase genes, is crucial for risk assessment and the development of an inventory of critical substitutions for molecular surveillance. Our findings provide timely and evidence-based insights that strengthen molecular surveillance efforts and improve our understanding of the evolutionary dynamics of emerging influenza A(H5N1) viruses.

## Methods

### Cells and viruses

Madin-Darby canine kidney (MDCK) (International Reagent Resource (IRR), Manassas, VA) and African green monkey kidney (Vero) cell lines (ATCC) were cultured in DMEM medium supplemented with 10% fetal bovine serum (FBS) and 100 U/ml penicillin-streptomycin. MDCK-SIAT1 cells (ATCC), which overexpress α2,6-linked sialic acid, were cultured in standard MDCK cell culture medium supplemented with 1 mg/mL geneticin (Thermo Fisher Scientific Inc.). Normal human bronchial epithelial (NHBE) cells (Lonza) and the human airway epithelial cell line Calu-3 (ATCC) were cultured and subsequently seeded onto Transwell inserts in 12-well plates (Corning) under air-liquid interface conditions as described previously^35^.

The original isolate (A/British Columbia/PHL-2032/2024, BC/24, identification number: EPI_ISL_19548836 in Global Initiative on Sharing All Influenza Data (GISAID)), propagated in MDCK cells (designated the C2 stock), was kindly provided by the Public Health Agency of Canada. The original stock virus, diluted 10^5^ to 10^6^-fold, was subsequently passaged either directly in MDCK-SIAT1 cells overexpressing α2,6-linked sialic acids (designated the C2S1 stock) or first in MDCK cells followed by an additional passage in MDCK-SIAT1 cells (designated the C2C1S1 stock). Both the C2S1 and C2C1S1 stocks of three different dilutions were sequenced using the Illumina iSeq NGS platform, and the average frequency of each variant at the HA-190 position was reported for each stock. The C2S1 stock contained predominantly the HA-190D variant (D at 98.6% frequency; E at 1.4% frequency), whereas the C2C1S1 stock contained predominantly the HA-190E variant (E at 97.1% frequency; D at 2.9% frequency). These were therefore referred to as the HA-190D and HA-190E stock viruses, respectively. In both stocks, HA-226Q and PB2-627K were present at >99% frequency (variant-calling threshold >1%). Both stock viruses were tested by real-time PCR to confirm the absence of contamination with other subtypes of IAV. Viral titers were determined by standard plaque formation assay and expressed as log_10_ PFU/mL. Additionally, NGS analysis confirmed that, aside from the difference at HA position 190, the two stock viruses shared identical consensus sequences. All work involved with the BC/24 virus, including animal studies, was conducted in Biosafety Level 3 containment laboratories, including enhancements (BSL-3E).

### Recombinant HA expression and glycan binding

The ectodomain of the HA from the BC/24 virus was synthesized as a codon-optimized construct (Genscript Inc.). The construct was subcloned into the pAcGP67-B baculovirus transfer vector, in frame with an N-terminal baculovirus GP67 signal peptide, a C-terminal thrombin cleavage site, a T4 fibritin trimerization tag for generating functional trimers, and a His-Tag for purification^36^. The constructs for A/Switzerland/9715293/2013 and A/Vietnam/1203/2004 have been described elsewhere^12^. Protein expression was performed in Hi5 insect cells (Thermo Fisher Scientific Inc.). Secreted soluble trimeric HA protein was recovered from the culture supernatant by tangential flow filtration using a 30 kDa molecular weight cut-off membrane, followed by metal affinity chromatography and size exclusion chromatography.

Glycan array analyses were performed using slides obtained under contract from the Centers for Disease Control and Prevention by James Paulson at The Scripps Research Institute (La Jolla, CA). Recombinant HA glycan microarray analyses were performed as described previously^37^. Briefly, 15 µg of recombinant HA-antibody complexes were mixed with mouse anti-6x-His-Tag-monoclonal antibody-Alexa Fluor 488 (Thermo Fisher Scientific Inc.) and anti-mouse-IgG-Alexa Fluor 488 (Thermo Fisher Scientific Inc.) in a molar ratio of 4:2:1, respectively. These mixtures were incubated for 1 h on ice, then diluted to 0.5 ml with a phosphate-buffered saline buffer containing 2% (wt/vol) bovine serum albumin before incubating on the microarray slides in a humidified chamber at 4 °C for 1.5 h. Subsequently, slides were washed by successive rinses in PBS with 0.05% (v/v) Tween 20, PBS, and deionized water. After drying under a nitrogen gas stream, the slides were then subjected to imaging. Fluorescence intensities were detected using an Innoscan 1100AL scanner (Innopsys, USA) and analyzed using MAPIX software (Innopsys, USA). A list of the glycans used in these experiments is included in Supplementary Table 2.

### HA genomic analysis

All available H5 IAV HA sequences up to December 29, 2025, were retrieved from the GISAID database. Duplicated entries and laboratory-derived recombinant viruses were excluded. The remaining HA sequences were categorized based on the virus name, host and passage history information. HA sequences with no clear host information and whose passage could not be clearly categorized were excluded. The remaining HA nucleotide sequences were aligned using the MAFFT^38^, and only sequences with complete nucleotide sequences at the three nucleotide positions coding for HA-190 were retained for further analysis. The frequency of various amino acids at the HA-190 position was reported. A total of 44,505 H5 HA sequences were included in the final analysis. The dataset can be accessed from GISAID using EPI_SET identifier EPI_SET_260106da (10.55876/gis8.260106da).

### Viral growth kinetics in NHBE, Calu-3, and MDCK cells

NHBE and Calu-3 cells cultured under air-liquid interface conditions in Transwell inserts, and MDCK cells cultured in 6-well plates, were inoculated with either the BC/24 HA-190D or HA-190E stock virus at a multiplicity of infection (MOI) of 0.01 for 1 h and subsequently washed three times with phosphate buffered Saline (PBS). NHBE and Calu-3 cells were then returned to air-liquid interface culture conditions, and 200 µL of culture medium was applied to the apical surface of each well for 15 min before being collected for viral titer determination at 2, 24, 48, and 72 h p.i. For MDCK cells grown on 6-well plates, 200 µL of infected cell culture supernatant was collected from each well at the same time points. All collected specimens were stored at −80 °C until subsequently being divided for either viral titration by standard plaque assay in MDCK cells or for viral RNA extraction and genomic analysis by NGS.

### Ferret infection

All animal studies were approved by the Institutional Animal Care and Use Committee of the Centers for Disease Control and Prevention (CDC) and conducted in accordance with their guidelines at an AAALAC International-accredited animal facility, and are reported in agreement with ARRIVE guidelines^39^. Two groups of male ferrets (*n* = 6 per group) (Mustela putorius furo, Triple F Farms, Sayre, PA, USA) of 20–30 months of age were used. All animals tested negative for circulating influenza viruses by hemagglutination inhibition (HAI) assays using a World Health Organization (WHO) kit^40^ (IRR, Manassas, VA). Ferrets in each group were anesthetized with ketamine (10–25 mg/kg) and xylazine (2–5 mg/kg) based on ferret body weight and inoculated intranasally with 100 PFU of either the BC/24 HA-190D or HA-190E stock virus diluted in PBS (day 0 p.i). Following inoculation, ferrets were monitored daily for clinical signs, body temperature rise, and weight loss. Additionally, throat swabs and nasal wash specimens were collected daily. On days 3 and 4 p.i., three ferrets from each group were humanely euthanized with Beuthanasia injection by the intracardiac route, and various tissues, swab specimens (throat and rectal), and nasal washes were collected and stored at -80 °C for subsequent viral titration and viral genomic analysis.

### Viral RNA extraction, amplification and NGS

Stock viruses, cell culture supernatants collected from inoculated NHBE, Calu-3, and MDCK cells, ferret nasal washes, and throat and rectal swabs were inactivated with AVL buffer (Qiagen) and viral RNAs were subsequently extracted using QIAamp Viral RNA kit (Qiagen). For ferret tissues, samples were submerged in RNAprotect Tissue Reagent (Qiagen) overnight at 4 °C, inactivated in RLT Plus buffer (Qiagen), and total RNA was then extracted using RNeasy Plus kit (Qiagen). Extracted RNA was reverse transcribed, and viral genomes were subsequently amplified using IAV universal primers and the Superscript III one-step RT-PCR system (Thermo Fisher Scientific), as previously described^41^. Amplicons were processed for sequencing using the Illumina Nextera DNA Flex library prep kit on the iSeq 100 (Illumina) platform. Raw reads were trimmed using BBDUK to remove adapters, low-quality reads (*Q* < 30), or short reads (<40 bp), and were subsequently aligned to the BC/24 virus genome in Geneious Prime (Version 2020.2.4). Variant identities at HA position 190 of the BC/24 viral genome were reported for specimens with a minimum sequencing depth >30×. The raw FASTQ files for these specimens, as well as for stock viruses, have been deposited in the National Center for Biotechnology Information (NCBI) Sequence Read Archive (SRA) under the BioProject accession number PRJNA1418753.

### pH thresholds for HA fusion activation

The pH threshold for HA fusion activation of each species of stock virus was evaluated by a syncytium formation assay as previously described^42^. Briefly, Vero cells were infected with either BC/24 HA-190D or HA-190E stock viruses overnight and subsequently incubated with low-pH fusion buffers (pH 5.4-6.0 in 0.1-unit increments) at 37 °C for 5 min to induce syncytium formation, followed by incubation with Vero cell culture medium for an additional 3 hours. The cells were subsequently stained with anti-NP antibody (clone A1, A3 blend; EMD Millipore) after being fixed with 3% paraformaldehyde and then visualized by immunofluorescence microscopy. The fusion threshold was defined as the highest pH value at which 50% or more syncytia were achieved among the infected cells. The average pH threshold for fusion activation from three independent experiments was reported.

### Statistical analysis

Statistical differences in viral titers between BC/24 HA-190D and BC/24 HA-190E virus inoculated cells or ferrets at each time point p.i. were analyzed by two-way ANOVA with Sidak’s multiple comparisons test upon confirming normality by the Shapiro–Wilk test in Graphpad Prism (version 10.5).

## Supplementary information

Supplementary information

Supplementary information

## Data Availability

NGS data have been deposited in the National Center for Biotechnology Information (NCBI) Sequence Read Archive (SRA) under the BioProject accession number PRJNA1418753. HA. sequence analysis dataset can be accessed from GISAID using EPI_SET identifier EPI_SET_260106da (10.55876/gis8.260106da). The raw data for Figs. 2–4 are in the Source data file.
