# Supplementary material for: Hemagglutinin E190D substitution in clade 2.3.4.4b A(H5N1) influenza virus reduces receptor binding and viral fitness
Source: Npj Viruses. 2026 May 15;4:36. doi: 10.1038/s44298-026-00197-2 (PMC13434642; doi:10.1038/s44298-026-00197-2)
Supplement: Supplementary file 1 [file 44298_2026_197_MOESM1_ESM.pdf]

Supplementary Figure 1.

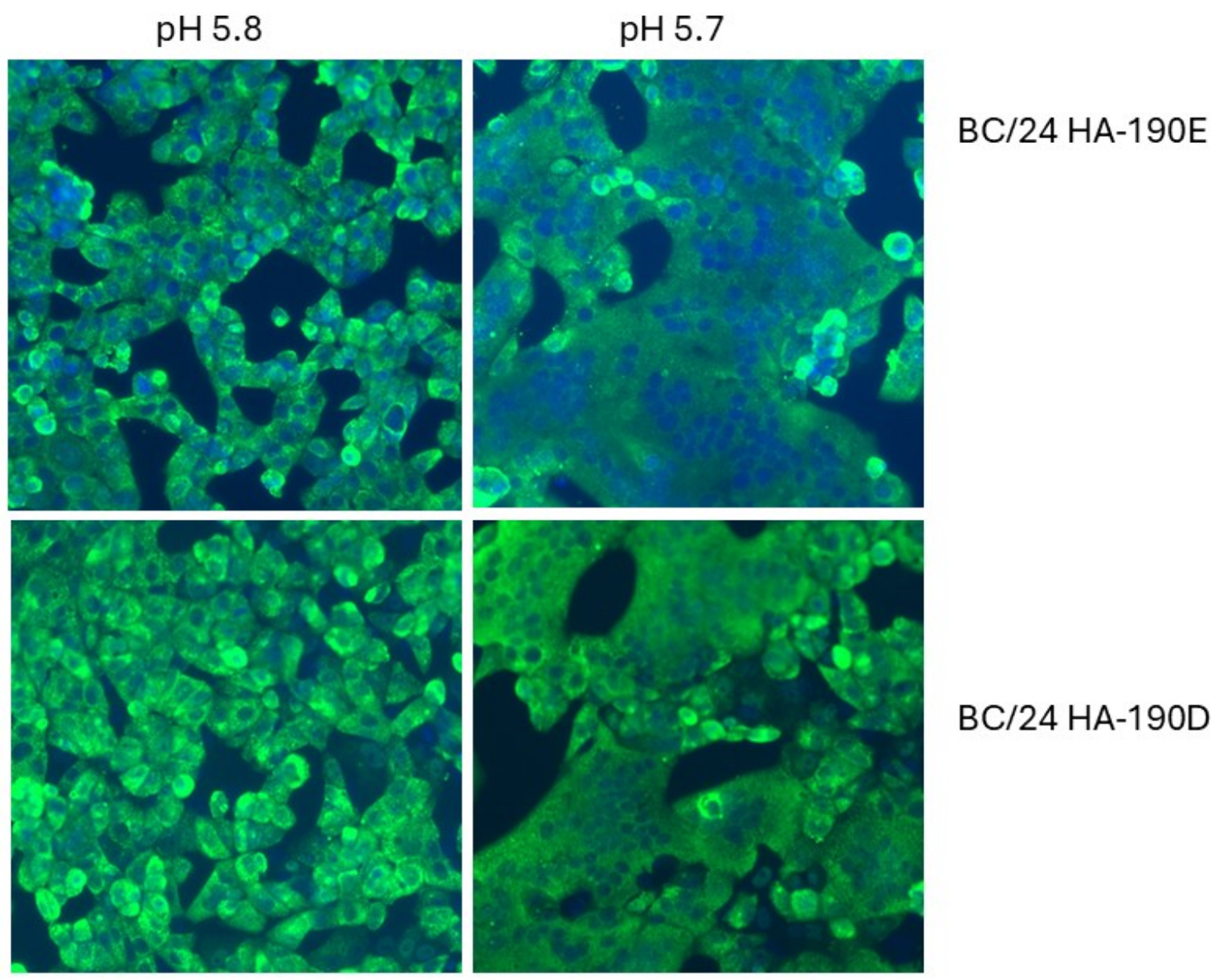

Supplementary Figure 1. pH threshold for HA-mediated fusion of BC/24 HA-190D and BC/24 HA-190E viruses. Vero cells were infected with either BC/24 HA-190D or HA-190E virus overnight, followed by induction of syncytium formation using low pH fusion buffer. At three hours post fusion induction, cells were fixed and stained with anti-NP antibody and visualized by immunofluorescence microscopy. The fusion threshold for both viruses was determined to be pH 5.7, as evidenced by syncytium formation in > 50% of infected cells, whereas no such fusion was observed at pH 5.8.

Supplementary Table 1. Diversity of amino acids at position 190 in the hemagglutinin proteins of H5 subtype of influenza viruses by host origin and passage history

Host	Avian					Human					Other Mammals				Environment‡‡	Insect§§
Passage	Original‡	Cell§	Egg¶	Mixed#	Unknown**	Original	Cell	Egg	Mixed	Unknown	Original	Cell	Egg	Unknown	NA	NA
E	17044	199	3610	5	15913	130	66	262	10	346	5094	36	89	445	994	2
V	98				19			1		1						
X*	45		1		11	4				3	35				1	
G	21														1	
A	1				3					1						
I	1															
N	1															
S			1													
K					5											
H					1											
P					1											
T					1											
D	1					1	1									

* Codons containing ambiguity characters were translated as 'X". Among these sequences, ten contained resolvable nucleotides, represented by IUPAC codes indicating two possible nucleotides. For these ten sequences, all potential amino acids at this position were evaluated, and none corresponded to aspartic acid (D). The remaining sequences included at least one undetermined nucleotide (N), rendering the amino acid at this position indeterminate.

‡Original: original clinical specimens from patients or original swabs from animals.

§Cell: Isolates were propagated and isolated in mammalian cells.

¶Egg: Isolates were propagated and isolated in eggs.

#Mixed: Isolation history included both cell and egg passages.

**Unknown: Passage history information is not provided.

‡‡ Environment: refers to specimens collected from environment, feces, surface swabs, and air samples.

§§ Insect: refers to specimens collected from blow flies, or beetles.

Supplementary Table 2: A list of glycans included in glycan array analyses

Chart #	Structure	Description
1	α-Neu5Ac–Sp8	α-Neu5Ac
2	α-Neu5Ac–Sp11	α-Neu5Ac
3	β-Neu5Ac-Sp8	β-Neu5Ac
4	Neu5Acα2-3(6-O-Su)Galβ1-4GlcNAcβ-ethyl-NH2	α2-3 so4
5	Neu5Acα2-3Galβ1-3(6OSO3)GalNAcα-Sp8	α2-3 so4
6	Neu5Acα2-3Galβ1-4(6OSO3)GlcNAcβ-Sp8	α2-3 so4
7	Neu5Acα2-3Galβ1-4(Fucα1-3)(6OSO3)GlcNAcβ–Sp8	α2-3 so4
8	Neu5Acα2-3Galβ1-3(6OSO3)GlcNAc-Sp8	α2-3 so4
9	Neu5Acα2-3Galβ1-3(Neu5Acα2-3Galβ1-4)GlcNAcβ-Sp8	di-sialoside
10	Neu5Acα2-3Galβ1-3(Neu5Acα2-3Galβ1-4GlcNAcβ1-6)GalNAc–Sp14	di-sialoside
11	Neu5Acα2-3Galβ1-4GlcNAcβ1-2Manα1-3(Neu5Acα2-3Galβ1-4GlcNAcβ12Manα1-6)Manβ1-4GlcNAcβ1-4GlcNAcβ-Sp12	α2-3 biantennary
12	NeuAcα(2-3)-Galβ(1-4)-GlcNAcβ(1-3)-Galβ(1-4)-GlcNAcβ(1-2)-Manα(1-3)-[NeuAcα(2-3)-Galβ(1-4)-GlcNAcβ(1-3)-Galβ(1-4)-GlcNAcβ(1-2)-Manα(1-6)]-Manβ(1-4)-GlcNAcβ(1-4)-GlcNAcβ-Asn-NH2	α2-3 biantennary
13	Neu5Acα2-3Galβ-Sp8	α2-3
14	Neu5Acα2-3Galβ1-3GalNAcα-Sp8	α2-3
15	Neu5Acα2-3Galβ1-3GlcNAcβ–Sp0	α2-3
16	Neu5Acα2-3Galβ1-3GlcNAcβ–Sp8	α2-3
17	Neu5Acα2-3Galβ1-4Glcβ–Sp0	α2-3
18	Neu5Acα2-3Galβ1-4Glcβ–Sp8	α2-3
19	Neu5Acα2-3Galβ1-4GlcNAcβ–Sp0	α2-3
20	Neu5Acα2-3Galβ1-4GlcNAcβ–Sp8	α2-3
21	Neu5Acα2-3GalNAcβ1-4GlcNAcβ-Sp0	α2-3
22	Neu5Acα2-3Galβ1-4GlcNAcβ1-3Galβ1-4GlcNAcβ-Sp0	α2-3
23	Neu5Acα2-3Galβ1-3GlcNAcβ1-3Galβ1-4GlcNAcβ-Sp0	α2-3
24	Neu5Acα2-3Galβ1-4GlcNAcβ1-3Galβ1-4GlcNAcβ1-3Galβ1-4GlcNAcβ– Sp0	α2-3
25	Neu5Acα2-3Galβ1-4GlcNAcβ1-3Galβ1-3GlcNAcβ-Sp0	α2-3
26	Neu5Acα2-3Galβ1-3GalNAc–Sp14	α2-3
27	Neu5Acα2-3Galβ1-4(Fucα1-3)GlcNAcβ1-6(Galβ1-3)GalNAc–Sp14	α2-3 fucosylated
28	Neu5Acα2-3Galβ1-3(Fucα1-4)GlcNAcβ–Sp8	α2-3 fucosylated
29	Neu5Acα2-3Galβ1-4(Fucα1-3)GlcNAcβ–Sp0	α2-3 fucosylated
30	Neu5Acα2-3Galβ1-4(Fucα1-3)GlcNAcβ–Sp8	α2-3 fucosylated
31	Neu5Acα2-3Galβ1-4(Fucα1-3)GlcNAcβ1-3Galβ-Sp8	α2-3 fucosylated
32	Neu5Acα2-3-Galβ1-3(Fucα1-4)GlcNAcβ1-3Galβ1-4(Fucα1-3)GlcNAcβ-Sp0	α2-3 fucosylated
33	Neu5Acα2-3Galβ1-4(Fucα1-3)GlcNAcβ1-3Galβ1-4(Fuca1-3)GlcNAcβ-Sp0	α2-3 fucosylated
34	Neu5Aca2-3Galβ1-4(Fuca1-3)GlcNAcβ1-3Galβ1-4(Fuca1-3)GlcNAcβ1-3Galβ1-4(Fuca1-3)GlcNAcβ-Sp0	α2-3 fucosylated

35	Neu5Acα2-3(GalNAcβ1-4)Galβ1-4GlcNAcβ-Sp0	α2-3 internal
36	Neu5Acα2-3(GalNAcβ1-4)Galβ1-4GlcNAcβ-Sp8	α2-3 internal
37	Neu5Acα2-3(GalNAcβ1-4)Galβ1-4Glcβ–Sp0	α2-3 internal
38	Neu5Acα2-3(Galβ1-3GalNAcβ1-4)Galβ1-4Glcβ-Sp0	α2-3 internal
39	Neu5Acα2-3(Fucα1-2Galβ1-3GalNAcβ1-4)Galβ1-4Glcβ-Sp0	α2-3 internal
40	Neu5Acα2-3(Fucα1-2Galβ1-3GalNAcβ1-4)Galβ1-4Glcβ-Sp9	α2-3 internal
41	Neu5Acα2-6Galβ1-4[6OSO3]GlcNAcβ-Sp8	α2-6 so4
42	Neu5Acα2-6Galβ1-4GlcNAcβ1-2Manα16(Galβ1-4GlcNAcβ1-2Manα1-3)Manβ1-4GlcNAcβ1-4GlcNAcβ-Sp12	α2-6 branched
43	Neu5Acα2-6Galβ1-4GlcNAcβ1-2Manα1-3(Neu5Acα2-6Galβ1-4GlcNAcβ1-2Manα1-6)Manβ1-4GlcNAcβ1-4GlcNAcβ-Sp12	α2-6 biantenary
44	NeuAcα(2-6)-Galβ(1-4)-GlcNAcβ(1-3)-Galβ(1-4)-GlcNAcβ(1-2)-Manα(1-3)-[NeuAcα(2-6)-Galβ(1-4)-GlcNAcβ(1-3)-Galβ(1-4)-GlcNAcβ(1-2)-Manα(1-6)]-Manβ(1-4)-GlcNAcβ(1-4)-GlcNAcβ-Sp12	α2-6 biantenary
45	Neu5Acα2-6Galβ1-4GlcNAcβ1-3Galβ1-4GlcNAcβ1-3Galβ1-4GlcNAcβ1-2Manα1-3(Neu5Acα2-6Galβ1-4GlcNAcβ1-3Galβ1-4GlcNAcβ1-3Galβ1-4GlcNAcβ1-2Manα1-6)Manβ1-4GlcNAcβ1-4GlcNAcβ-Sp12	α2-6 biantenary
46	Neu5Acα2-6Galβ1-4GlcNAcβ1-3Galβ1-4GlcNAcβ(1-3)(Neu5Acα2-6Galβ1-4GlcNAcβ1-3Galβ1-4GlcNAcβ1-6)GalNAca-Sp14	α2-6 biantenary
47	NeuAcα2-6Galβ1-4GlcNAcβ1-3[NeuAcα2-6Galβ1-4GlcNAcβ1-6]GalNAca-SP14	α2-6 biantenary
48	Neu5Acα2-6GalNAcα-Sp8	α2-6
49	Neu5Acα2-6Galβ-Sp8	α2-6
50	Neu5Acα2-6Galβ1-4Glcβ-Sp8	α2-6
51	Neu5Acα2-6Galβ1-4Glcβ-ethyl-NH2	α2-6
52	Neu5Acα2-6Galβ1-4GlcNAcβ-Sp0	α2-6
53	Neu5Acα2-6Galβ1-4GlcNAcβ-Sp8	α2-6
54	Neu5Acα2-6GalNAcβ1-4GlcNAcβ-Sp0	α2-6
55	NeuAcα2-6Galβ1-4GlcNAcβ1-3GalNAcα-Thr-NH2	α2-6
56	Neu5Acα2-6Galβ1-4GlcNAcβ1-3Galβ1-4GlcNAcβ-Sp0	α2-6
57	Neu5Acα2-6Galβ1-4GlcNAcβ1-3Galβ1-4GlcNAcβ1-3GalNAca-Sp14	α2-6
58	Neu5Aca2-6Galβ1-4GlcNAcβ1-3Galβ1-4GlcNAcβ1-3Galβ1-4GlcNAcβ-Sp0	α2-6
59	Neu5Acα2-6Galβ1-4GlcNAcβ1-3Galβ1-4(Fucα1-3)GlcNAcβ1-3Galβ1-4(Fucα1-3)GlcNAcβ-Sp0	α2-6 fucosylated
60	Neu5Acα2-6(Galβ1-3)GlcNAcβ1-4Galβ1-4Glcβ-Sp10	α2-6 internal
61	Neu5Acα2-6(Galβ1-3)GalNAca-Sp14	α2-6 internal
62	Neu5Acα2-6Galβ1-4GlcNAcβ1-6(Galβ1-3)GalNAca-Sp14	α2-6 internal
63	NeuAcα2-6Galβ1-4GlcNAcβ1-3Galβ1-4GlcNAcβ1-6(Galβ(1-3)GalNAca-Sp14	α2-6 internal
